# Effects of carbon ion beam-induced mutagenesis for the screening of RED production-deficient mutants of *Streptomyces coelicolor* JCM4020

**DOI:** 10.1371/journal.pone.0270379

**Published:** 2022-07-14

**Authors:** Masaomi Yanagisawa, Shumpei Asamizu, Katsuya Satoh, Yutaka Oono, Hiroyasu Onaka

**Affiliations:** 1 Graduate School of Agricultural and Life Sciences, The University of Tokyo, Bunkyo, Tokyo, Japan; 2 Collaborative Research Institute for Innovative Microbiology (CRIIM), The University of Tokyo, Bunkyo, Tokyo, Japan; 3 Department of Radiation-Applied Biology Research, Takasaki Advanced Radiation Research Institute, Quantum Beam Science Research Directorate, National Institutes for Quantum Science and Technology, Takasaki, Gunma, Japan; University of Tsukuba, JAPAN

## Abstract

*Streptomyces lividans* TK23 interacts with mycolic acid-containing bacteria (MACB), such as *Tsukamurella pulmonis* TP-B0596, and this direct cell contact activates its secondary metabolism (e.g., the production of undecylprodigiosin: RED). Here, we employed carbon (^12^C^5+^) ion beam-induced mutagenesis to investigate the signature of induced point mutations and further identify the gene(s) responsible for the production of secondary metabolites induced by *T*. *pulmonis*. We irradiated spores of the *Streptomyces coelicolor* strain JCM4020 with carbon ions to generate a mutant library. We screened the RED production-deficient mutants of *S*. *coelicolor* by mixing them with *T*. *pulmonis* TP-B0596 on agar plates, identifying the red/white phenotype of the growing colonies. Through this process, we selected 59 RED-deficient mutants from around 152,000 tested spores. We resequenced the genomes of 16 mutants and identified 44 point mutations, which revealed the signatures induced by ^12^C^5+^-irradiation. Via gene complementation experiments, we also revealed that two genes—glutamate synthase (*gltB*) and elongation factor G (*fusA*)—are responsible for the reduced production of RED.

## Introduction

*Streptomyces* spp. is an industrially important Gram-positive filamentous actinobacterium that produces bioactive natural products (secondary metabolites) used as antibiotics, pesticides, and for anticancer treatments [1,2]. Each strain contains more than 20–40 putative biosynthetic gene clusters for secondary metabolites [3,4]. Although they are considered to have the potential to produce those genome-encoded cryptic secondary metabolites, the number of metabolites detectable in general laboratory culture conditions is still limited [4,5].

We previously reported that a group of mycolic acid-containing bacteria (MACB; e.g., *Tsukamurella pulmonis* TP-B0596) affected the secondary metabolism of actinomycetes in combined-culture [6]. Combined-culture is a co-culture method that involves the combination of actinomycetes and MACB to activate the production of secondary metabolites by actinomycetes. MACB are also a group of actinomycetes containing specific long-chain fatty acids (C_30_–C_60_ mycolic acids) on the cell wall [7,8]. MACB (e.g., *T*. *pulmonis* TP-B0596) can activate the production of undecylprodigiosin (RED) and actinorhodin (ACT) by *Streptomyces lividans* TK23, which are not produced by this strain under general laboratory culture conditions [6]. In fact, *T*. *pulmonis* TP-B0596 was shown to be effective at inducing the production of a diverse range of secondary metabolites by various actinomycetes [9–17], and this strategy was also found to be efficient for enhancing production during heterologous expression [18–20].

Studies have reported that the above-mentioned activation of RED and ACT did not occur through the provision of culture extracts or killed bacteria [6,21]. However, scanning electron microscopy (SEM) revealed that *S*. *lividans* and several MACB formed co-aggregates [21]. Meanwhile, co-aggregation and the production of RED and ACT were not observed upon the addition of killed MACB (e.g., killed by γ-ray irradiation or formaldehyde fixation) [21]. Therefore, it was suggested that the formation of co-aggregates generates continuous physical cell contact with living *T*. *pulmonis*, leading to activation of the production of RED and ACT by *S*. *lividans*.

A convenient strategy for bacterial mutagenesis is ultraviolet (UV) irradiation or chemical treatment with *N*-methyl-*N*′-nitro-*N*-nitrosoguanidine (NTG). UV radiation is an ubiquitous and potent DNA-damaging mutagen [22]. The most common type of DNA damage caused by UV is covalent linkages between two adjacent pyrimidines, resulting in the generation of a cyclobutane pyrimidine dimer (CPD) and pyrimidine(6–4)pyrimidone photoproducts (6-4PP) [23]. Failure to detect and repair such DNA lesions is a major cause of mutagenesis. This chemical coupling results in the mutation signature of C-to-T transitions [22]. UV is also involved in the production of reactive oxygen species (ROS) from cellular O_2_, which has been reported to be generated by activating cellular substances, such as riboflavin, tryptophan, and porphyrin [22]. ROS attack DNA and produce the mutagenic 8-oxo-7,8-dihydroguanine (8-oxoG) [24]. The signature of ROS-induced mutation is C-to-A transversion [24], which is less common but also present in the genomes of cells mutated by UV irradiation [22,25]. NTG induces mutations by alkylating purines and pyrimidines in DNA. The majority of mutations caused by NTG are C-to-T transitions, a signature similar to that of UV-induced mutations [26]. Although UV and NTG are convenient and widely used mutagens, their signatures can cause a bias that limits the ability to generate random mutations evenly across the whole of the bacterial genome.

Ionizing radiation induces DNA double-strand breaks (DSBs), a particularly serious form of DNA damage that is especially deleterious to cells. Its mutagenic effects depend on high linear energy transfer (LET). Ion beams have been used for plant breeding and shown to have highly lethal and mutagenic effects, causing a low number of mutations in a locus but instead large-scale genomic variations [27]. Recently, the use of ion beam mutagenesis technology has been expanded to the breeding of various microorganisms [28,29]. Genomic analyses of the ion beam-induced mutants have been reported, clarifying the effects of ion beam mutagenesis on microorganisms such as *Escherichia coli* [30], *Bacillus subtilis* [31], and *Saccharomyces cerevisiae* [32]. It has been proposed that radiation doses that leave a surviving fraction of a population of 1%–10% are effective for achieving mutagenesis in microorganisms.^33^ Regarding the application of heavy-ion mutagenesis to *Streptomyces* spp., several examples that show the improved production of useful secondary metabolites have been reported [33–35]. However, because the number of applications using heavy-ion mutagenesis for bacteria is still limited, and the mutants have not been comprehensively characterized at the genome scale, there is still a need to understand the signature of mutations in the *Streptomyces* genome [28,29].

The purpose of this study was to obtain initial insight into the signatures of point mutations in *Streptomyces* species, which are GC-rich spore-forming actinobacteria, produced by heavy ion beam-induced mutagenesis at the genome scale. Two factors were considered to obtain mutants of interest prior to the study: application of a ^12^C^5+^ ion beam (surface LET, 107 keV/μm), which is known to be highly mutagenic among the different levels of LET for the breeding of microorganisms, and mutant selection from a high survival rate to reduce the multiple point mutations. In this study, mutants of *Streptomyces coelicolor* JCM4020 that exhibited the loss or reduction of RED production were screened from a pooled spore mutant library using combined culture with *T*. *pulmonis*. We also report that the sites of DNA mutation were identified and two genes were suggested to be responsible for the reduced production of RED in respective mutants by gene complementation study.

## Materials and methods

### Preparation of mutant library using carbon ion beams

*Streptomyces coelicolor* JCM4020 was used as a reference and its whole-genome nucleotide sequence was deposited in a public database with accession number AP025454. A total of 1×10^8^ spores of JCM4020 were transferred to a 1.5 ml tube from the freeze stock and 900 μl of 0.5×PBS with 20% glycerol was added. After centrifugation (3,000 rpm, 3 min, 4°C), the supernatant was discarded and 1 ml of 0.1×PBS was added to suspend the spores, followed by the application of 0.5 ml of sample on a mixed cellulose ester membrane (47 mm i.d., 0.2 μm) on the Petri dish (55 mm i.d.). After air-drying inside the flow cabinet, the Petri dish was covered by 7.5-μm-thick kapton polyimide film (Toray-Dupont) and ^12^C^5+^ ions (220 MeV, surface LET: 107 keV/μm) were irradiated by the AVF cyclotron at TIARA, QST, Takasaki. The sample was prepared twice. The first irradiation doses were 100 Gy, 500 Gy, and 1,000 Gy (n = 3 each), and the second irradiation doses were 10 Gy, 50 Gy, 100 Gy, and 200 Gy (n = 3 each). The irradiated spores were recovered by adding 0.5 ml of 20% glycerol solution and scraping the surface with a spoon. This step was repeated four times and the suspended spore solution was collected and centrifuged (3,000 rpm, 5 min, 4°C). After discarding the supernatant, 1 ml of 20% glycerol solution was added and the sample was stored at −80°C as the irradiated spore stock until use.

### Survival rates and aerial mycelium formation rates

The irradiated spore stock was further diluted as appropriate using sterilized H_2_O and 100 μl was applied on a tryptone soya broth agar plate. After incubation at 30°C for 3 days, the number of colony forming units (CFU) was counted. The survival rate was calculated by comparison to the 0 Gy sample, which was similarly treated but without irradiation. To obtain the rate of aerial mycelium formation, approximately 100 colonies were grown on each MS agar [36] plate and incubated at 30°C for 7 days. A visible white appearance of the colony was used to judge whether or not aerial mycelium had formed. Colonies forming aerial mycelium were counted and divided by the total number of inoculated colonies.

### Screening of mutants

Initial screening of the mutants was performed in two ways. (S1 and S2 Figs) For the first one, the cell stock of *T*. *pulmonis* TP-B0596 was diluted in sterilized H_2_O and 100 μl of this cell (about 1 × 10^5^ cells) suspension was applied to YGGS agar [21]. On the same plate, an irradiated spore stock was diluted to give approximately 100 colonies on a single agar plate and incubated at 30°C for 4–7 days. *T*. *pulmonis* grew in the bacterial lawn, where *S*. *coelicolor* colonies could also be found. Most of the *S*. *coelicolor* colonies were red in color, but colonies with weak or no coloring were selected. The *T*. *pulmonis* cells were removed by growing each picked colony directly on mixed cellulose ester membrane (0.45 μm). We used the feature that *S*. *coelicolor* filamentous cells can pass through a 0.45 μm membrane and reach the agar medium, but *T*. *pulmonis* with its larger size cannot. After incubation at 30°C for 3 to 7 days, the membrane was removed and further incubated for 2–7 days. The obtained filtered mutant cells were subcultured on new YGGS agar and incubated at 30°C for 3–5 days. The formed colonies were suspended in 20% glycerol solution and stored at −80°C until use.

In the second screening approach, we considered the possibility that irradiated spores contain damaged DNA and thus require a period of recovery to form a colony from a single spore. Growth with *T*. *pulmonis* from the beginning may thus affect the growth of the mutants and thus reduce their yield. On the YGGS agar plate, irradiated spore stock was applied to have approximately 60 colonies on a single agar plate and incubated at 30°C for 3 days. On the other YGGS plate, *T*. *pulmonis* was grown in the bacterial lawn by incubation at 30°C for 3 days. A metal cylinder covered by velour-surfaced material was used to stamp the agar culture of *T*. *pulmonis* and then stamped on the agar culture of *S*. *coelicolor* to inoculate *T*. *pulmonis* (similar to traditional colony hybridization in the molecular biology technique). After incubation at 30°C for 3 days, mutant colonies of *S*. *coelicolor* were picked and further isolated by the same methods as described above.

### Mutant phenotype examination under different culture conditions

The phenotype of the mutants obtained by the screening was first confirmed by dual culture with *T*. *pulmonis*. Two microliters of *T*. *pulmonis* cell stock was spotted on the agar plate and 2 μl of *S*. *coelicolor* mutant cell stock was spotted at a distance of 1 cm. The cells were incubated at 30°C and the production of RED in the area where the colonies contacted each other was visually examined in comparison to the phenotype of the wild-type strain as a control. The mutant with less or no productivity of RED was further grown on minimal medium [36] by spotting 2 μl of *S*. *coelicolor* mutant cell stock; the growth was then visually examined after incubation at 30°C for 7 days. The mutant that could grow on the minimal medium was further tested for the ability to form aerial mycelium. Two microliters of *S*. *coelicolor* mutant cell stock was used to inoculate Bennett’s maltose agar [36] and MS agar medium [36], followed by incubation at 30°C for 7 days. A visible white appearance of the colony was used to judge whether or not aerial mycelium had formed. The mutant forming aerial mycelium was further tested for the ability to produce RED under NaCl stress. Two microliters of *S*. *coelicolor* mutant cell stock was used to inoculate Bennett’s maltose with 1% NaCl. Production of RED under osmotic stress was examined by the visible red appearance of the colony.

### PacBio RSII sequencing of wild type, DNA library preparation, and MiSeq sequencing of mutants

Genomic DNA of the wild type and mutants was isolated by the CTAB protocol [36]. Single-molecule real-time sequencing (PacBio RSII) was used to determine the complete genome sequence of *S*. *coelicolor* JCM4020 by BGI (https://www.bgi.com). A library was prepared twice to generate 20 kb fragments and 0.3 and 0.9 Gb of sequence data were obtained. The obtained long read sequence data were assembled by Canu/Celera Genome Assembler [37] and a single contig was obtained. The CDS were deduced using the DFAST (https://dfast.ddbj.nig.ac.jp/) pipeline with the automatic annotation of gene function [38]. Briefly, 4671 CDS features were detected by MetaGeneAnnotator [39], 54 tRNA features were detected by Aragorn [40], 6 rRNA features were detected by Barrnap (https://github.com/tseemann/barrnap).

The genomes purified from the mutants and wild type were broken up into approximately 300 bp fragments by NEBNext^®^ dsDNA Fragmentase^®^. Agencourt AMPure XP (Beckman Coulter) was then used to purify these DNA fragments. Agilent Bioanalyzer 2100 with the High Sensitivity DNA kit (Agilent) was used to confirm the fragment size. The DNA library was prepared using NEBNext^®^ Ultra™ II DNA Library Prep Kit for Illumina^®^. Index primers were selected from NEBNext^®^ Multiplex Oligos for Illumina^®^ (Index Primers Sets 1 and 2, NEB #E7335, #E7500). The purity and yield of the generated DNA library were analyzed by Agilent Bioanalyzer 2100 with High Sensitivity DNA kit. MiSEQ (Illumina) with MiSeq Reagent Kit v3 (paired end, 75×2 cycles) was used for sequencing. The obtained short-read FASTA data were imported and analyzed using CLC Genomic Workbench software ver. 10 (Qiagen). After mapping the short reads to the reference genome sequences of JCM4020 wild type, nucleotide substitutions, insertions, and deletions were detected by comparison to the MiSEQ sequence data of the wild type obtained at the same time. Large deletions of the genome were searched manually. The identified point mutations were confirmed by Sanger sequencing of the PCR-amplified products.

### Gene complementation study

Using genomic DNA of the JCM4020 strain as a template, primers (S1 Table) were used to amplify the DNA fragments containing *gltB* (*sco2026* homolog) and *fusA* (*sco4661* homolog) by PCR. To consider the expression level in the original strain, the original promoter was used to express the gene. The cloning region containing the 5’-UTR of the genes was determined based on prediction of the polycistronic transcription unit. KOD Plus NEO (Toyobo) was used for the PCR, following the manufacturer’s protocol. The amplified 6.46 kb DNA fragment containing the region homologous to *sco2026-25* was digested by *Bam*HI and *Hin*dIII, and ligated into the corresponding site of the pTYM19t plasmid [41] to generate pTYM19-gltB. The amplified 4.64 kb DNA fragment containing the region homologous to *sco4659-62* was digested by *Eco*RI and *Hin*dIII, and ligated into the corresponding site of the pTYM19t plasmid to generate pTYM19-fusA. The standard protocol for a conjugation method using *E*. *coli* ET12567 (pUZ8002) was performed to introduce the plasmids into the spores of the JCM4020 strain [36]. The obtained mutants were selected using 20 μg/ml thiostrepton and applied for further study. To compare the responses among the wild type, mutant, and gene-complemented mutant, dual culture with *T*. *pulmonis* was performed on an agar plate. Spore stock solutions (2 μL, 2–3×10^5^ CFU) of mutant harboring empty vector (pTYM19t), mutant harboring the expression plasmid (pTYM19t-gltB), and JCM4020 wild type harboring empty vector (pTYM19t) were inoculated by spotting on YGGS agar medium. At the same time, cell stock solution (2 μL, 5×10^6^ CFU) of *T*. *pulmonis* was spotted on the agar medium. After the inoculation, the cells were grown at 30°C for 7 days and observed over time. The same procedure was carried out for the gene complementation of *fusA*.

## Results

### Genome sequence of *Streptomyces coelicolor* JCM4020

The genome size of *S*. *coelicolor* JCM4020 was 8,634,640 bp, comprising a single linear chromosome. JCM4020 did not contain any plasmids. Its GC content was 72.2 mol% and the number of coding sequences (CDS) was 7799. The identified CDS were almost identical to those of the model actinomycetes strain *S*. *coelicolor* A3(2) M145, which has a genome size of 8,667,507 bp, 72.1 mol% GC content, and 7877 CDS [42]. In this paper, the SCO numbers used for *S*. *coelicolor* A3(2) M145 gene and CDS designations are used for convenience. *S*. *coelicolor* JCM4020 was used in this experiment because this strain did not produce RED or ACT in the monoculture conditions using YGGS agar medium used in the screening or dual culture (Figs 2 and 3), in comparison to the model actinomycetes *S*. *coelicolor* A3(2) M145, which produced RED and ACT. This phenotypic character conferred an advantage to screen RED-deficient mutants in combined culture. Secondary metabolite gene clusters in the strain JCM4020 were predicted by antiSMASH [43] (S2 Table). The results showed that the contents of predicted secondary metabolite gene clusters were completely identical to those of *S*. *coelicolor* A3(2) M145.

### ^12^C^5+^ ion irradiation and dose-dependent survival rates

Mutagenesis by carbon ion beams was performed at TIARA (Takasaki Ion Accelerators for Advanced Radiation Application) in QST (National Institutes for Quantum Science and Technology) on different days. First, 100 Gy, 500 Gy, and 1,000 Gy of ^12^C^5+^ ions were irradiated (1^st^ irradiation in Table 1). The survival rates were measured by counting the colony forming units (CFU), which were then compared with those upon no irradiation, giving values of 68.8 ± 25.1% for 100 Gy, 7.4 ± 0.68% for 500 Gy, and 0.52 ± 0.10% for 1,000 Gy irradiation (Table 1). We also tested the formation of aerial mycelium in this step using the colony morphology as an indication. Overall, 99.9 ± 0.065% of the formed colonies generated aerial mycelium at 0 Gy, 93.6 ± 1.2% at 100 Gy, 63.3 ± 4.0% at 500 Gy, and 40.4 ± 16.2% at 1,000 Gy. Second, in line with the procedure used in the first irradiation, 10 Gy, 50 Gy, 100 Gy, and 200 Gy of ^12^C^5+^ ions were irradiated (2^nd^ irradiation in Table 1). The survival rates were 55.0 ± 3.5% at 10 Gy, 36.1 ± 8.6% at 50 Gy, 36.0 ± 5.5% at 100 Gy, and 13.0 ± 4.1% at 200 Gy (Table 1). The formation of aerial mycelium was also tested, showing rates of 99.9 ± 0.065% for 0 Gy, 99.3 ± 0.35% for 10 Gy, 99.3 ± 0.43% for 50 Gy, 98.4 ± 1.1% for 100 Gy, and 90.6 ± 2.3% for 200 Gy. Overall, the survival rate and aerial mycelium formation rate decreased depending on the irradiation dose, suggesting that the frequency of point mutations depends on the irradiation dose.

**Table 1:** Survival rates and aerial mycelium formation rate of the carbon ion-irradiated spores of *Streptomyces coelicolor* JCM4020.

Dose (Gy)	survival rate (%) for 1st irradiation samples	aerial mycelium forming rate (%) for 1st irradiation samples	survival rate (%) for 2nd irradiation samples	aerial mycelium forming rate (%) for 2nd irradiation samples
0	100	99.9 ± 0.065	100	99.9 ± 0.065
10			55.0 ± 3.5	99.3 ± 0.35
50			36.1 ± 8.6	99.3 ± 0.43
100	68.8 ± 25.1	93.6 ± 1.2	36.0 ± 5.5	98.4 ± 1.1
200			13.0 ± 4.1	90.6 ± 2.3
500	7.4 ± 0.68	63.3 ± 4.0		
1000	0.52 ± 0.10	40.4 ± 16.2		

### Screening of RED production-deficient mutants

**Fig 1:**
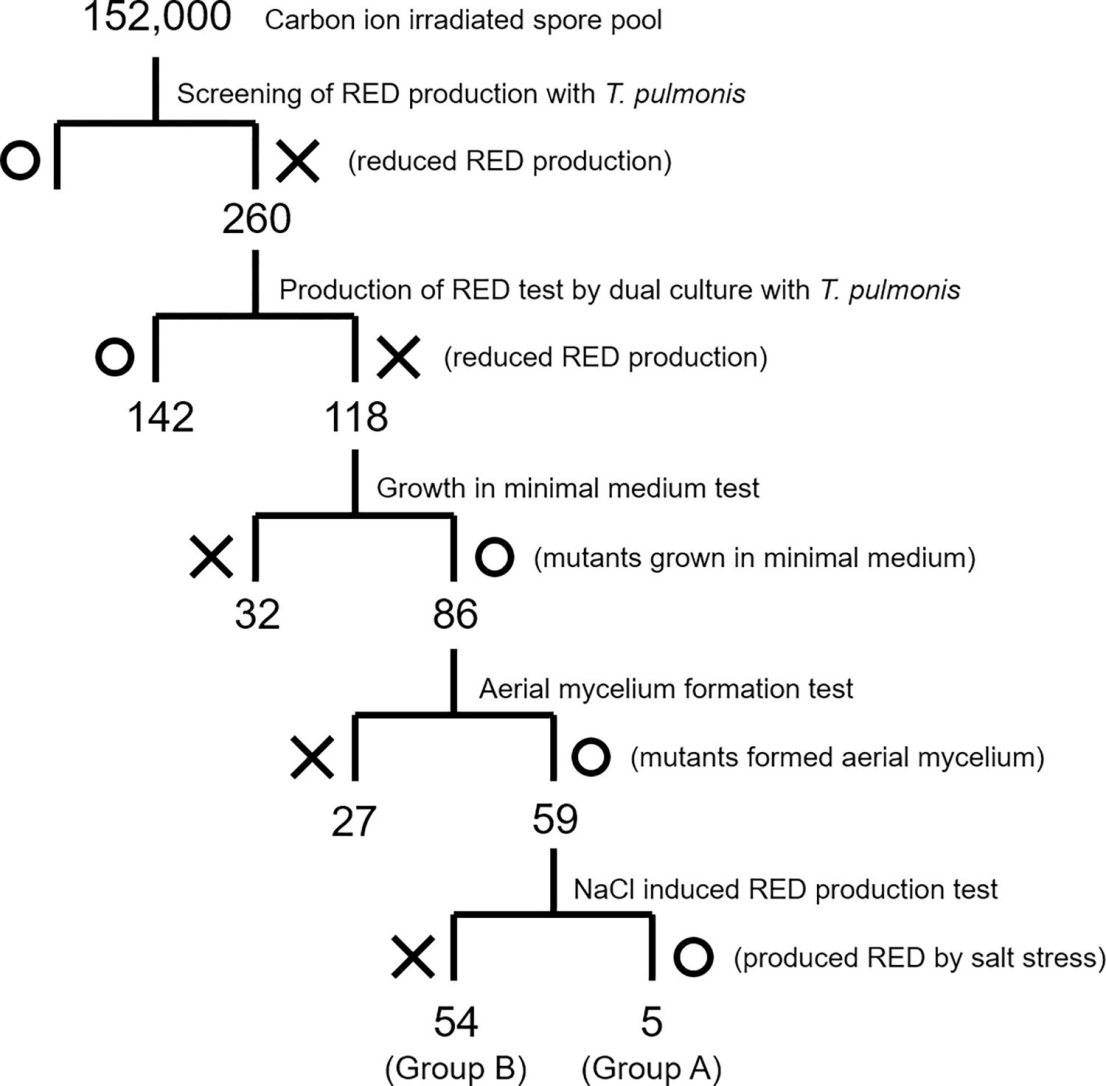
Screening of RED-deficient mutants of *S*. *coelicolor* JCM4020.

**Fig 2:**
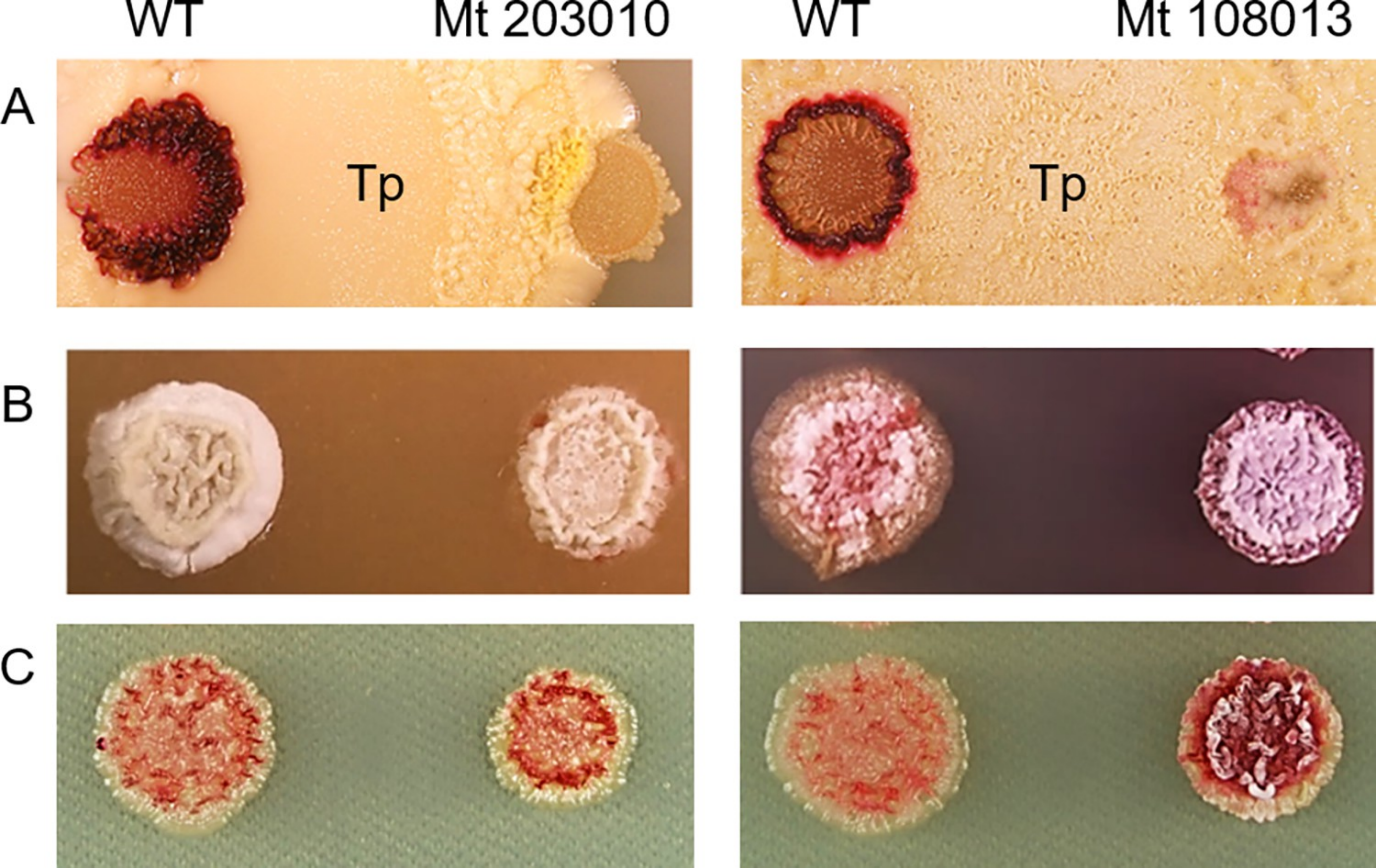
Phenotype of the mutants. A: Competitive dual culture among strain JCM4020 wild-type (WT) or Mutant with *T*. *pulmonis*. B: Formation of aerial mycelium. C: Production of RED under salt stress condition.

We chose mutant spore libraries from the first 100 Gy irradiation, second 100 Gy irradiation, and second 200 Gy irradiation for the screening of mutants, considering the survival rates. In total, we tested approximately 152,000 spores (based on estimation from the CFU) and selected 118 mutants (with a yield of 0.078%) that showed lower or lost production of RED upon incubation with *T*. *pulmonis* TP-B0596 (Figs 1, S1 and S2). The low yield (0.078%) of mutant may have been because we used the mutant spore library showing a relatively high survival rate (13.0%–68.8%). Radiation doses giving a surviving fraction of 1%–10% have been proposed for effective mutagenesis in microorganisms.^33^ The use of a sample with a lower survival rate may increase the number of mutants exhibiting abnormal RED production, but it may also increase multiple undesired mutations in the genome, leading to difficulty identifying the genes responsible for the phenotype. We then tested the growth on minimal medium and the formation of aerial mycelium for 118 mutants, 59 of which showed no deficiency (Fig 1). We excluded mutants that could not grow on the minimal medium from further analysis because they may exhibit specific auxotrophy by possessing a primary metabolism-related mutation that significantly affects growth, in turn affecting the secondary metabolism. We also excluded mutants that could not form aerial mycelium from further analysis at this point because the genes responsible for the bald phenotype have been extensively studied [44,45] and to reduce the rediscovery of already-characterized genes. There was a concern that the mutations would occur in the biosynthetic gene cluster of RED, which directly affect the production. The production of RED by *Streptomyces coelicolor* A3(2) was reported to be activated by the addition of NaCl [46]. We then tested the effect of salt stress by adding 1% NaCl to the medium because *S*. *coelicolor* JCM4020 did not produce RED on the tested normal medium in monoculture. Five mutants showed RED production comparable to that of the wild type in the salt-stressed condition (Figs 1 and 2). Although the mechanism of its induction by salt stress was unclear, it was confirmed that at least these mutants do not contain significant lesions in the biosynthetic gene cluster of RED.

### Identified point mutations

Subsequently, we performed genome re-sequencing to identify the point mutations in the genome of screened mutants to investigate the signature of induced point mutations and further identify the gene(s) responsible for the production of secondary metabolites. Overall, 16 mutants were selected, namely, 3 out of 5 mutants (in group A, Fig 1) that produce RED in salt stress conditions and 13 out of 54 mutants (in group B, Fig 1) that form aerial mycelium, which showed a consistent phenotype (Figs 1 and S3–S7). The genome was resequenced using a next-generation sequencer, MiSEQ (Illumina). Upon comparison with the sequence of the wild type, we identified 58 point mutations (Tables 2 and 3). The identified point mutations were confirmed by further Sanger sequencing. Six mutants (Mt 202001, Mt 202004, Mt 202007, Mt 205011, Mt 208014, Mt 20980) contained an identical C-to-A transversion at genomic position 4,593,233 bp and the insertion of a G at genomic position 4,565,012 bp (Table 2). Moreover, two mutants (Mt 209010, Mt 203013) contained the insertion of a C at genomic position 4,564,124 bp (Table 2). It is unlikely for an identical point mutation to be induced at the same position, so these 14 (6+6+2) point mutations were considered to have arisen naturally during cell growth for spore preparation. Therefore, the other 44 point mutations were considered to have been induced by the carbon ions. Upon 100 Gy of irradiation, 14 point mutations from 7 mutants (average 2.0 point mutations/mutant) were found, while upon 200 Gy of irradiation, 30 point mutations from 9 mutants (average 3.3 point mutations/mutant) were found. The number of mutations showed a certain correlation with the irradiation dose. Among these 44 mutations, there were 31 base substitutions, 5 insertions, and 8 deletions (Table 3). The 44 point mutations were distributed relatively evenly across the whole genome. Meanwhile, we did not detect any large-scale genomic variations, such as large deletions, translocations, or inversions in the carbon ion-irradiated *S*. *coelicolor* JCM4020. Because the sequenced mutants in this study were screened from multiple screening steps and further selected from the mutants showing a stable phenotype, we assumed that mutants containing large-scale genomic variations that may involve loss of genes essential for survival and aerial mycelium formation were excluded through the multiple screening steps.

**Table 2:** Point mutations found in the RED production-deficient mutants of *S*. *coelicolor* JCM4020.

No.	Mutant ID	Genomic region	Position of nucleotide mutation	Position of amino acid mutation	Category of mutation	Putative function of deduced protein	Gene locus tag	SCO number	identity (%)
1	Mt 108013	4,857,490	670T>C	Ser223Pro	M	integrase	JCM4020_44710	SCO4340	99.6
		2,302,923	597delC	Pro200FS	F	glutamate synthase large subunit (GltB)	JCM4020_21750	SCO2026	99.9
		588,274	974G>T	Gly325Val	M	hypothetical protein	JCM4020_05430	SCO0562	99.2
2	Mt 203010	6,277,489	930C>A		S	riboflavin kinase / FMN adenylyltransferase	JCM4020_58030	SCO5711	100.0
		5,170,097	147delC	Thr50>FS	F	elongation factor G (FasA)	JCM4020_47790	SCO4661	100.0
3	Mt 107004	5,207,800	484_486delGAC	del_Asp162	D	50S ribosomal protein L4 (Rpl4)	JCM4020_48150	SCO4703	100.0
4	Mt 209020	5,566,038	insAGCTT			UTR			
		5,386,522	1038delC	Arg347>FS	F	hypothetical protein	JCM4020_49890	SCO4877	99.1
		5,107,024	195G>T		S	hypothetical protein	JCM4020_47210	SCO4587	99.5
5	Mt 204003	8,370,142	843C>T		S	oxidoreductase	JCM4020_75780	SCO7572	99.7
		6,935,841	379_381delCGC	del_Arg127	D	hypothetical protein	JCM4020_63500	SCO6192	99.7
		2,858,275	555G>T	Gln185His	M	chitinase precursor	JCM4020_26770	SCO2503	98.7
		2,828,233	G>T			UTR			
		2,240,012	783C>T		S	hydrolase	JCM4020_21170	SCO1968	99.6
		305,254	1303T>C	Phe435Leu	M	membrane protein (putative)	JCM4020_02940	SCO0308	99.8
		272,610	91_92delCG	Arg31>FS	F	hypothetical protein	JCM4020_02670	SCO0281	100.0
6	Mt 204030	7,850,278	C>A			UTR			
		5,940,690	19G>C	Glu7Gln	M	large Ala/Glu-rich protein	JCM4020_55130	SCO5397	99.9
		5,581,527	2324G>A	Trp775*	N	bifunctional protein	JCM4020_51760	SCO5064	99.0
		4,692,036	160G>C	Ala54Pro	M	hypothetical protein	JCM4020_43070	SCO4177	97.4
		1,885,408	103_104insCC	Tyr36>FS	F	hypothetical protein	JCM4020_17890	SCO1647	100.0
7	Mt 202001	4,593,233	C>A			UTR			
		4,565,012	1316_1317insG	Val440>FS	F	membrane protein (SarA)	JCM4020_41980	SCO4069	100.0
		3,131,571	1054G>A	Glu352Lys	M	methylmalonic acid semialdehyde	JCM4020_29060	SCO2726	100.0
		2,726,723	104T>C	Phe35Ser	M	MarR family transcriptonal regulator	JCM4020_25700	SCO2398	99.3
8	Mt 202004	5,148,481	G>T			UTR			
		4,727,540	352G>T	Glu118*	N	GntR family transcriptional regulator	JCM4020_43460	SCO4215	100.0
		4,593,233	C>A			UTR			
		4,565,012	1316_1317insG	Val440>FS	F	membrane protein (SarA)	JCM4020_41980	SCO4069	100.0
9	Mt 202007	6,013,594	22 T>C	Ser8Pro	M	two-component system response regulator	JCM4020_55720	SCO5455	100.0
		4,593,233	C>A			UTR			
		4,565,012	1316_1317insG	Val440>FS	F	membrane protein (SarA)	JCM4020_41980	SCO4069	100.0
		3,043,516	C>G			UTR			
		1,791,652	G>A			UTR			
		1,602,792	1344_1345CC>TT	Leu449Phe	M	acetoacetate-CoA ligase	JCM4020_15290	SCO1393	99.7
10	Mt 205011	4,593,233	C>A			UTR			
		4,565,012	1316_1317insG	Val440>FS	F	membrane protein (SarA)	JCM4020_41980	SCO4069	100.0
		3,444,416	795C>T		S	preprotein translocase subunit SecA	JCM4020_31820	SCO3005	99.9
		2,493,601	712G>A	Ala238Thr	M	lipoyl synthase	JCM4020_23420	SCO2194	99.7
11	Mt 208014	4,593,233	C>A			UTR			
		4,565,012	1316_1317insG	Val440>FS	F	membrane protein (SarA)	JCM4020_41980	SCO4069	100.0
		2,981,624	G>A			UTR			
		2,708,647	A>G			UTR			
12	Mt 209008	4,771,848	1209C>T		S	hydrolytic protein	JCM4020_43880	SCO4257	99.8
		4,593,233	C>A			UTR			
		4,565,012	1316_1317insG	Val440>FS	F	membrane protein (SarA)	JCM4020_41980	SCO4069	100.0
		2,803,393	C>T			UTR			
		2,498,314	93delG	Val32>FS	F	integrase (putative)	JCM4020_23470	(SCO3997)	29.5
13	Mt 203013	8,232,911	81delG	Glu27>FS	F	cytochrome P450 (fragment)	JCM4020_74450	SCO7444	99.4
		7,751,457	411G>A		S	ABC transporter ATP-binding protein	JCM4020_40060	SCO7008	99.8
		4,564,124	428_429insC	Ala144>FS	F	membrane protein (SarA)	JCM4020_41980	SCO4069	100.0
		3,244,900	427C>T	Leu142Phe	M	hypothetical protein	JCM4020_30080	SCO2827	100.0
		604,854	1747_1748insCC	Arg584>FS	F	dihydroxyacetone kinase	JCM4020_05610	SCO0580	98.8
14	Mt 209010	4,564,124	428_429insC	Ala144>FS	F	membrane protein (SarA)	JCM4020_41980	SCO4069	100.0
		3,885,136	C>A			UTR			
15	Mt 106003	6,495,294	876_877insATC	Ile292_Pro293insIle	I	RedP	JCM4020_59860	SCO5888	99.1
16	Mt 201001	6,511,887	2639C>G	Pro880Arg	M	RedH	JCM4020_59940	SCO5896	99.7
		4,073,556	130_131insGG	Leu44>FS	F	hypothetical protein	JCM4020_37140	SCO3619	97.3

No.1-3: Three out of five mutants which produced RED in salt containing medium (group A). The other 13 out of 54 sequenced mutants were from group B. No. 7–14: Eight mutants containing a point mutation in *sarA* gene. No. 15–16: Two mutants containing a point mutation in RED biosynthetic gene. Abbreviations in category of mutation; M: Amino acid substitution; F: Flame shift mutation; N: Nonsense mutation; S: Silent mutation; I: Insertion of amino acid; D: Deletion of amino acid. Identities (%): Amino acid sequence identities between CDS form strain JCM4020 and A3(2) M145.

**Table 3:** Type of point mutations found in the RED-deficient mutants of *S*. *coelicolor* JCM4020 by carbon ion irradiation.

type	point mutations	number
substitution	G/C to A/T	21
	A/T to G/C	5
	G/C to C/G	4
	CC >TT	1
(total)		31
deletion	gelG/C	5
	delA/T	0
	delCG	1
	delGCG	1
	delTCG	1
(total)		8
insertion	insCC	3
	insGAT	1
	insAGCTT	1
(total)		5

### Identification of amino acid mutations

Amino acid mutations in the CDS caused by point mutations were identified. Among the identified 44 point mutations, at the amino acid level they caused 13 missense mutations, 2 nonsense mutations, 9 frameshifts, 1 amino acid insertion, 2 amino acid deletions, and 7 silent mutations, while the remaining 10 were in noncoding regions (Table 2). Overall, 27 amino acid mutations were considered to affect the function of the encoded protein. Two mutants (Mt 106003, Mt 201001) contained mutations in RED biosynthetic genes (*redH* and *redP*, respectively), indicating that these mutations directly cause deficiency of RED biosynthesis (Table 2, S8 Fig). As described previously, naturally arising point mutations involving the insertion of a G at 4,565,012 bp in six mutants and the insertion of a C at 4,564,124 bp in two mutants cause frameshift in the SarA (SCO4069) homolog originally found in *Streptomyces coelicolor* A3(2) [47]. As the deletion of *sarA* causes a defect in RED production [47], it was considered that *sarA* mutants were accumulated by our screening method. Finally, six mutants containing a total of 14 amino acid mutations were considered for the identification of candidate genes involved in the production of RED induced by *T*. *pulmonis* stimulation.

### Gene complementation for phenotypic recovery

**Fig 3:**
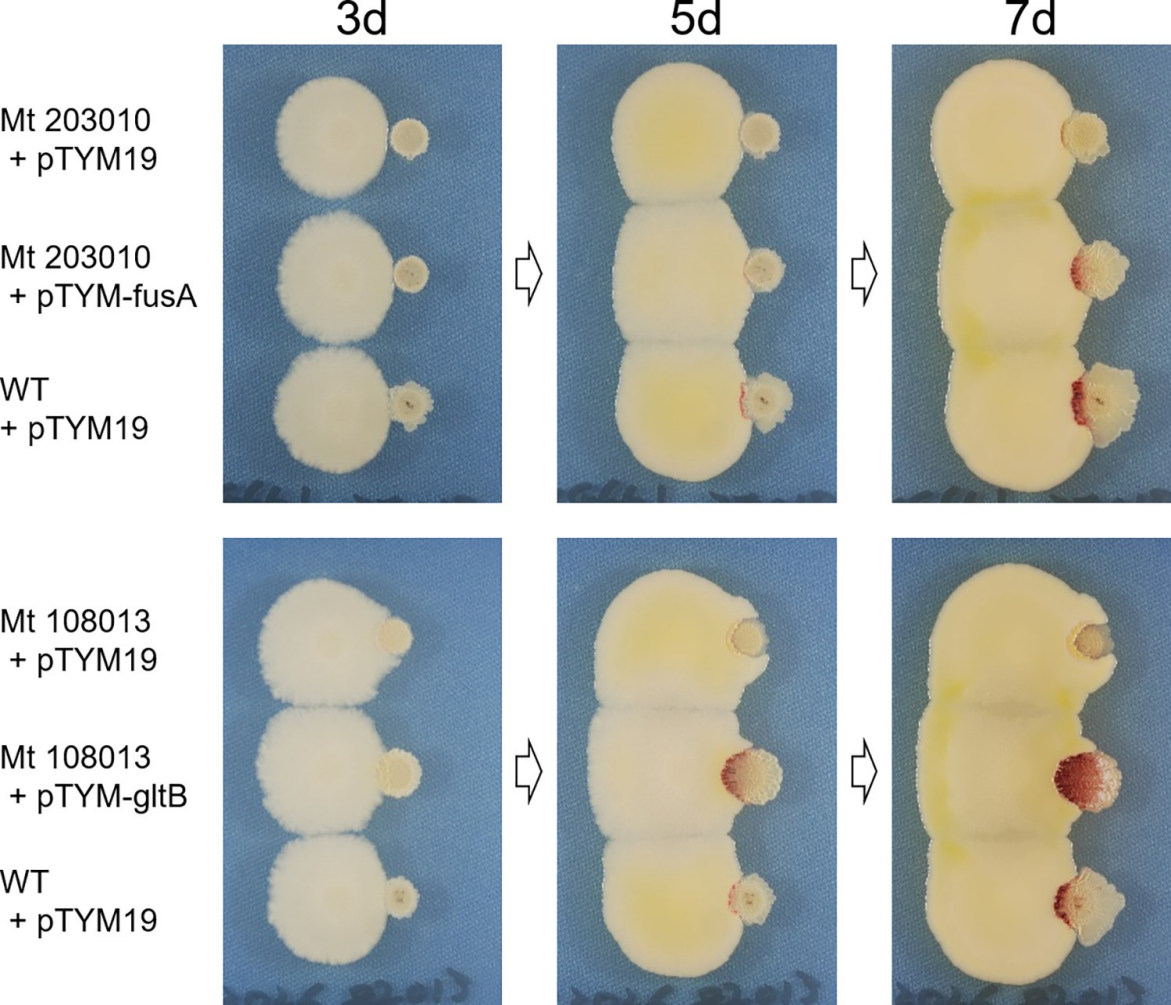
Gene complementation of *gltB* in Mt 108013 and *fusA* in Mt 203010 rescued the productivity of RED in condition interacting with *T*. *pulmonis*.

Three of the 16 genome-resequenced mutants (Mt 108013, 203010, Mt 107004) formed aerial mycelium and produced RED in agar medium containing 1% NaCl in Bennett’s maltose at levels comparable to those in the wild type (Figs 2 and S3). We performed gene complementation for Mt 108013 and Mt 203010. Mt 108013 possessed a mutation in glutamine synthase (GltB, SCO2026 homolog), and Mt 203010 possessed one in elongation factor G (EF-G) (FusA, SCO4661 homolog) (Table 2). In Mt 108013, C^597^ of the *gltB* gene was deleted, resulting in frameshift of the amino acid sequence (S8 Fig). The DNA fragment containing the *gltB* gene was amplified by PCR from the wild-type JCM4020 and cloned into the pTYM19t vector. The pTYM19t-gltB vector was introduced into Mt 108013 and the production of RED was compared with that of the wild type. Mt 108013 complemented by pTYM19-gltB recovered the capacity to produce RED in combined-culture with *T*. *pulmonis*, compared with Mt 108013 with an empty vector (Figs 3 and S9). The strain harboring pTYM19-gltB showed rather higher productivity of RED than the wild type in dual culture (Fig 3). It is assumed that this occurred because we introduced the gene *in trans* (by ΦC31 integrase), which may affect the expression level, resulting in incomplete restoration of the phenotype. In Mt 203010, C^147^ of the *fusA* gene was deleted, which also resulted in frameshift of the amino acid sequence. (S10 Fig) The DNA fragment containing the *fusA* gene with 5’-UTR was amplified by PCR from the wild-type JCM4020 and cloned into the pTYM19t vector. The pTYM19t-fusA vector was introduced into Mt 203010 and its production of RED was compared with that of the wild type. Mt 203010 complemented by pTYM19-fusA recovered the production of RED in combined-culture with *T*. *pulmonis*, compared with Mt 203010 with an empty vector (Figs 3 and S11).

## Discussion

In this study, we evaluated the mutagenesis signature generated in *Streptomyces* spp. by a carbon ion beam. To screen the mutants, samples irradiated with 100 and 200 Gy were chosen based on the associated survival rates. Overall, the survival rate was dose-dependent, but showed some variation between the first and second irradiations. The ^12^C^5+^ ion beam can transfer energy to samples according to the Bragg curve. On the sample surface, the level of irradiation was 107 keV/μm and the Bragg peak gave the highest energy (more than 600 keV/μm) which is right before the ion progression terminates. The penetration range of ^12^C^5+^ ion in water is 1110 μm. Therefore, it was considered that the inconsistent thickness of the sample (overlapping spores in this case) may have affected the LET between the sample and thus affected the survival rate [29].

By resequencing the genomes of 16 mutants, we found 58 point mutations. Fourteen of those were predicted to be naturally arising mutations. Therefore, 44 mutations were predicted to have been induced by the ion beam, which included 18 transitions, 13 transversions, 5 insertions, and 8 deletions. Among the 31 nucleotide substitutions found in this study, 13 mutations (41.9%, 13/31) were G/C-to-A/T transitions (Table 3). This relatively low frequency of G/C-to-A/T transitions gave a different point mutation ratio overall from the pattern of UV irradiation (usually gave more than 70%). Five other A/T-to-G/C transitions (16.1%, 5/31) formed a signature not known to be produced by other methods of mutagenesis. For the other mutations, 13 (41.9%, 13/31) were transversions, involving a change from a purine to a pyrimidine base or vice versa (Table 3). Among these 13 transversions, 9 (29.0%, 9/31) were C/G-to-A/T mutations and 4 were G/C-to-C/G mutations. G to T is a signature point mutation induced by reactive oxygen species (ROS). ROS can be generated by UV irradiation, as well as heavy-ion irradiation [48]. The results indicate that the majority of mutation types [71.0%, (13+9)/31] are similar to the mutations induced by UV (C to T) or ROS (C to A). Additionally, when we mapped the 44 point mutations on the genome, the distribution was found to be relatively even across the whole genome. Theoretically, a carbon ion beam can attack DNA and introduce double-strand breaks. The capacity to repair such breaks might be less effective in bacteria, although in *Streptomyces coelicolor* A3(2) Ku protein (SCO5309) was shown to be responsible for non-homologous end joining (NHEJ) [49]. In addition to SNPs, we found that 29.5% (13/44) of the mutations were indels, including five insertions and eight deletions. Because there has been little study of the effects of carbon ions on mutagenesis in *Streptomyces* bacteria, further accumulation of findings may be needed to reveal the detailed signature of carbon ion mutagenesis. Taken together, our findings indicate specific features of mutagenesis produced by irradiation with carbon ions, involving a different mutational signature from those in conventional mutagenesis methods.

Using the screening methods applied in this study, we hoped to obtain mutants with mutations in genes involved in the response to and/or regulation of the stimulation provided by *T*. *pulmonis*. As described above, naturally occurring point mutations involving the insertion of a G at 4,565,012 bp in six mutants and the insertion of a C at 4,564,124 bp in two mutants caused frameshift in the SarA (SCO4069) homolog [47]. The *sarA* (*sco4069*) gene product is annotated as a hypothetical membrane protein and was also found in a transposon mutagenesis study, which showed that its inactivation had negative effects on the production of RED and actinorhodin [50,51]. Therefore, it was suggested that the RED production deficiency of the eight mutants had been caused by inactivation of the *sarA* gene. Although the function of SarA in the production of RED and ACT is unknown, it was demonstrated that interaction with *T*. *pulmonis* also cannot recover this production and suggested that SarA may be involved in the common regulatory mechanism, leading to the production of RED upon *T*. *pulmonis* stimulation. The *gltB* gene was found to be responsible for the phenotype of reduced production of RED. GltB is part of GOGAT, a conserved primary metabolism enzyme that generates two molecules of glutamate from glutamine and 2-oxoglutarate [52]. Glutamate plays a central role in nitrogen metabolism, including the synthesis of proline or serine that become direct precursors for the biosynthesis of RED [53]. Therefore it was speculated that limitation of the precursor supply caused the reduced production of RED associated with *gltB* mutation. Although further investigation is required, the addition of NaCl to the medium achieved the production of RED in this *gltB* mutant, suggesting that the stimulatory mechanisms for the production of RED mediated by salt stress and by combined culture with *T*. *pulmonis* differ. In addition to the above findings, the *fusA* gene was found to be responsible for the phenotype of reduced RED production. The *fusA* gene encodes EF-G, which is widely conserved in organisms and an essential factor for ribosome translocation [54]. *S*. *coelicolor* possesses an ortholog of the *fusA* gene (*fusB* gene; *sco6589* or *sco1528*), which might complement the function of FusA, resulting in the mutation not being lethal to the cell. It was speculated that deletion of the principal EF-G (*fusA*) in Mt 203010 may delay translation and therefore affect the production of RED. In Mt 107004, deletion of CGA (486–488) in the *rpl4* gene was observed. As a factor responsible for ribosome translation, *fusA* causes reduced RED production, and the mutation in this ribosomal protein was also considered to be involved in the RED-deficient phenotype by affecting its translation. Considering the functions of GltB and FusA, they may not have direct relationships with the system regulating the response to *T*. *pulmonis* stimulation that leads to RED biosynthesis, but it was considered that the disturbance of essential metabolism associated with their mutations can alter the production of RED. In addition to the *gltB* and *fusA* genes, *sarA* that is involved in the production of RED was rediscovered. As mutations in these genes reduced the basal production of RED in monoculture, it was confirmed that these genes are indeed responsible for the basal production of RED, but again they may not be relevant to the response to and/or regulatory mechanism of *T*. *pulmonis* stimulation. However, we have not examined all of the mutations by complementation to identify the genes involved in the observed phenotype of reduced or lost production of RED. Further identification of other mutated genes may provide new insight into the mechanism behind the production of RED, and deepen our understanding of the responses to and/or regulatory mechanisms of the stimulation by MACB, including *T*. *pulmonis* TP-B0596.

## Supporting information

S1 Fig Summary of method to screen RED deficient mutants of *S. coelicolor* JSM4020 (First screening method). Details are described in Material and methods.
(TIF)

S2 Fig Summary of method to screen RED deficient mutants of *S. coelicolor* JSM4020 (Second screening method). Details are described in Material and methods.
(TIF)

S3 Fig Phenotype of the mutants (Mt 108013, Mt 203010, Mt 107004). A: dual culture of strain JCM4020 wild type (Wt) or respective Mutant (Mt) with *Tsukamurella pulmonis* (Tp) grown on YGGS medium, day 5th. B: Wt and Mt grown on Bennett’s maltose, day 11th. C: Wt and Mt grown on Bennett’s maltose+1% NaCl, day 6th.
(TIF)

S4 Fig Phenotype of the mutants (Mt 209020, Mt 204003, Mt 204030). A: dual culture of strain JCM4020 wild type (Wt) or respective Mutant (Mt) with *Tsukamurella pulmonis* (Tp) grown on YGGS medium, day 5th. B: Wt and Mt grown on Bennett’s maltose, day 11th. C: Wt and Mt grown on Bennett’s maltose+1% NaCl, day 6th.
(TIF)

S5 Fig Phenotype of the mutants (Mt 202001, Mt 202004, Mt 202007, Mt 205011). A: dual culture of strain JCM4020 wild type (Wt) or respective Mutant (Mt) with *Tsukamurella pulmonis* (Tp) grown on YGGS medium, day 5th. B: Wt and Mt grown on Bennett’s maltose, day 11th. C: Wt and Mt grown on Bennett’s maltose+1% NaCl, day 6th.
(TIF)

S6 Fig Phenotype of the mutants (Mt 208014, Mt 209008, Mt 203013, Mt 209010). A: dual culture of strain JCM4020 wild type (Wt) or respective Mutant (Mt) with *Tsukamurella pulmonis* (Tp) grown on YGGS medium, day 5th. B: Wt and Mt grown on Bennett’s maltose, day 11th. C: Wt and Mt grown on Bennett’s maltose+1% NaCl, day 6th.
(TIF)

S7 Fig Phenotype of the mutants (Mt 106003, Mt 201001). A: dual culture of strain JCM4020 wild type (Wt) or respective Mutant (Mt) with *Tsukamurella pulmonis* (Tp) grown on YGGS medium, day 5th. B: Wt and Mt grown on Bennett’s maltose, day 11th. C: Wt and Mt grown on Bennett’s maltose+1% NaCl, day 6th.
(TIF)

S8 Fig Mutation caused frameshift in GltB and indicated to inactivate the function. (TIF)

S9 Fig Complementation of *gltB* (*sco2026*) gene in the Mt 108013 restore the production of RED. (TIF)

S10 Fig Mutation caused frameshift in FusA and indicated to inactivate the function. (TIF)

S11 Fig Complementation of *fusA* (*sco4661*) gene in the Mt 203010 restore the production of RED. (TIF)

S1 Table Primer sequences used in this study. (TIF)

S2 Table Result of antiSMASH (ver. 6.0.1) for prediction of secondary metabolite biosynthetic gene cluster in the *Streptomyces coelicolor* JCM4020. (TIF)
