# Supplementary figures and images for: Effects of carbon ion beam-induced mutagenesis for the screening of RED production-deficient mutants of Streptomyces coelicolor JCM4020
Source: PLoS One. 2022 Jul 14;17(7):e0270379. doi: 10.1371/journal.pone.0270379 (PMC9282665; doi:10.1371/journal.pone.0270379)

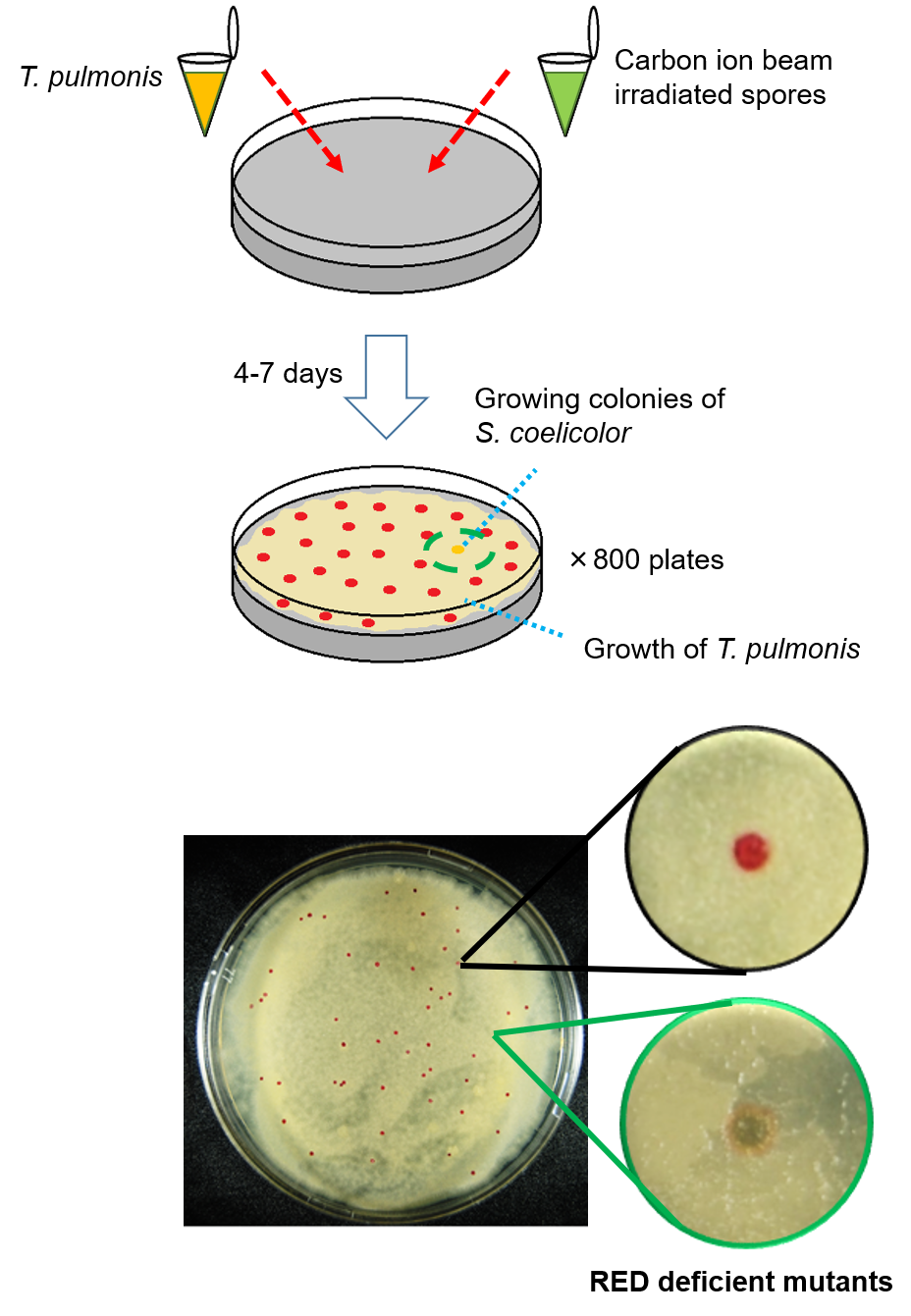

Supplement: S1 Fig — Details are described in Material and methods. (TIF) [file pone.0270379.s001.tif]

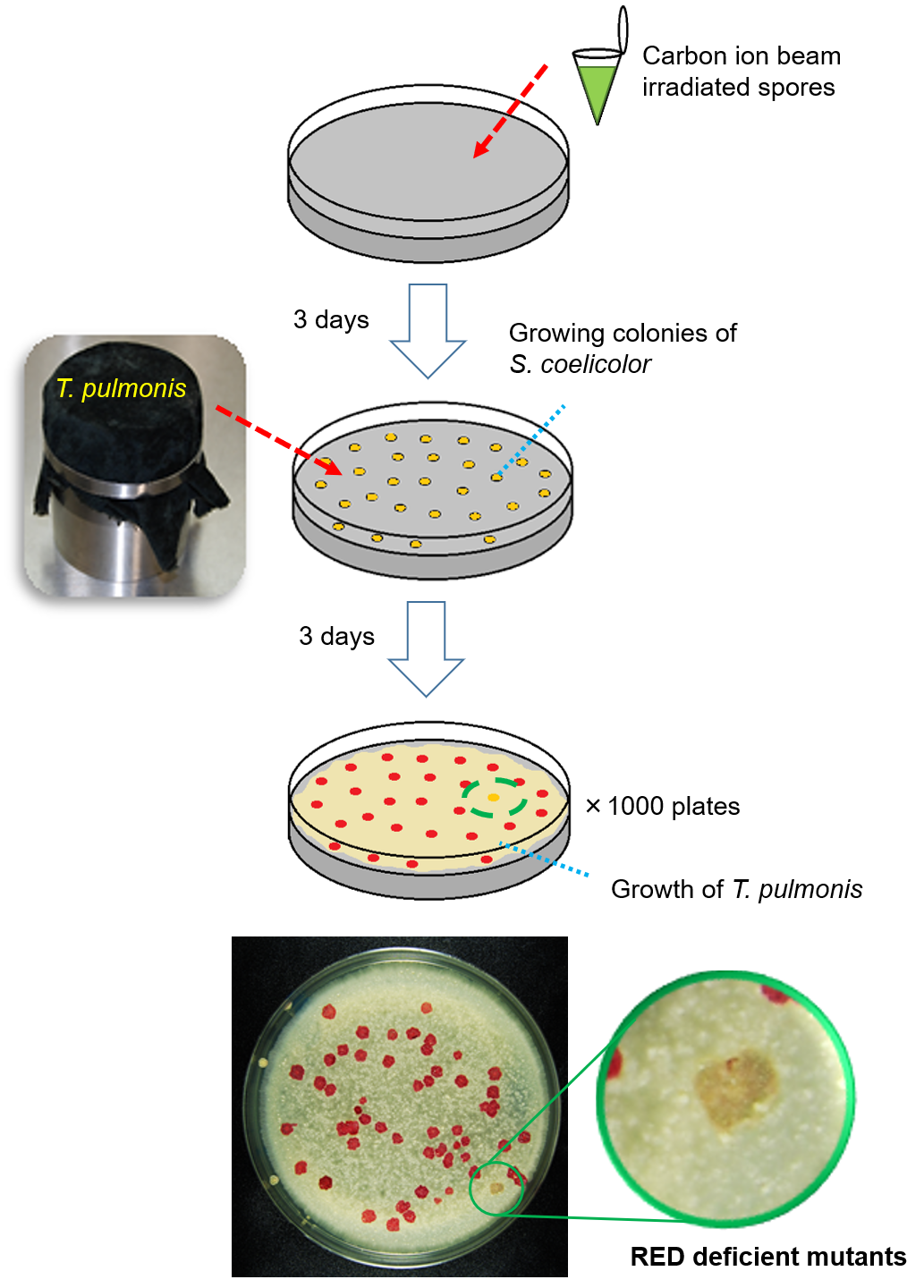

Supplement: S2 Fig — Details are described in Material and methods. (TIF) [file pone.0270379.s002.tif]

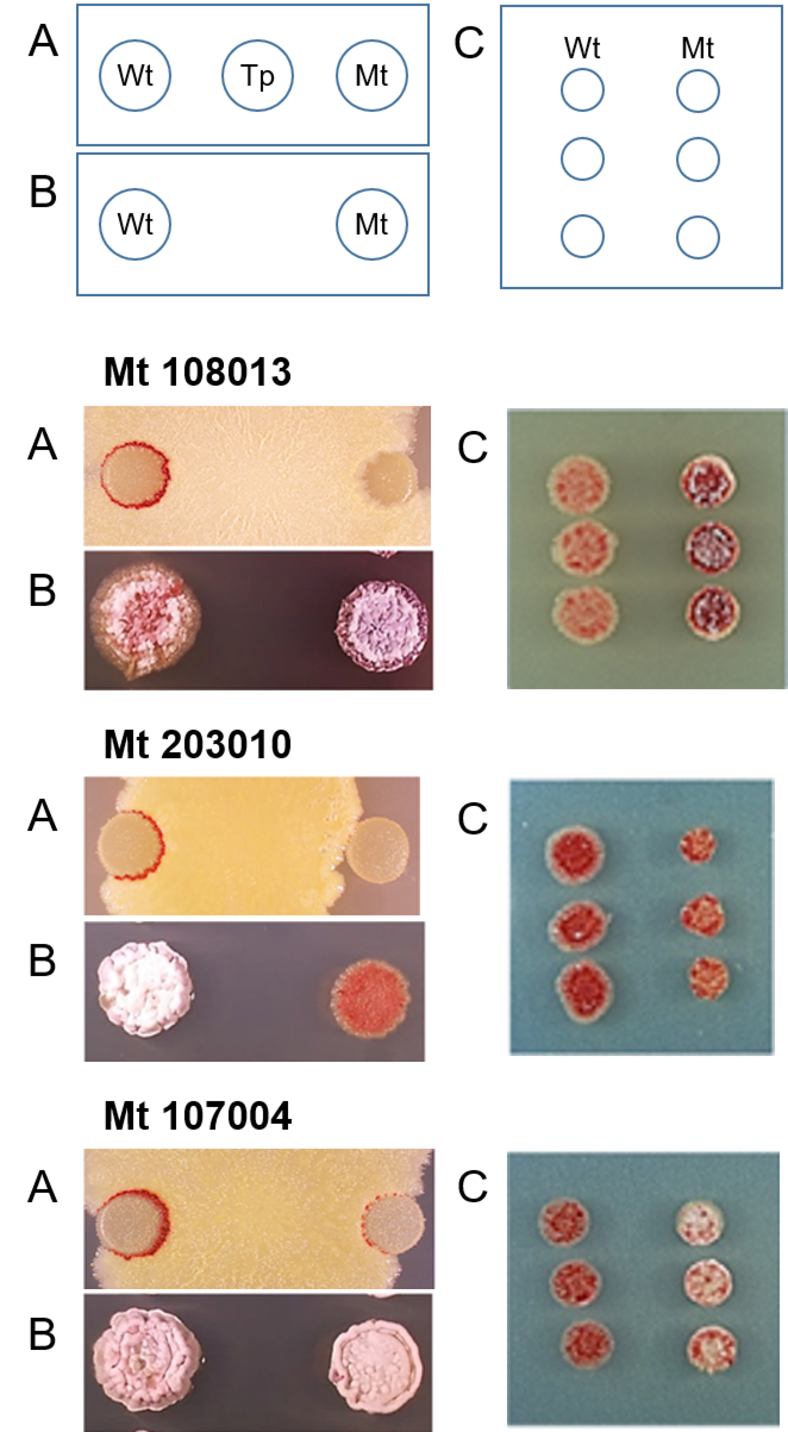

Supplement: S3 Fig — A: dual culture of strain JCM4020 wild type (Wt) or respective Mutant (Mt) with Tsukamurella pulmonis (Tp) grown on YGGS medium, day 5th. B: Wt and Mt grown on Bennett’s maltose, day 11th. C: Wt and Mt grown on Bennett’s maltose+1% NaCl, day 6th. (TIF) [file pone.0270379.s003.tif]

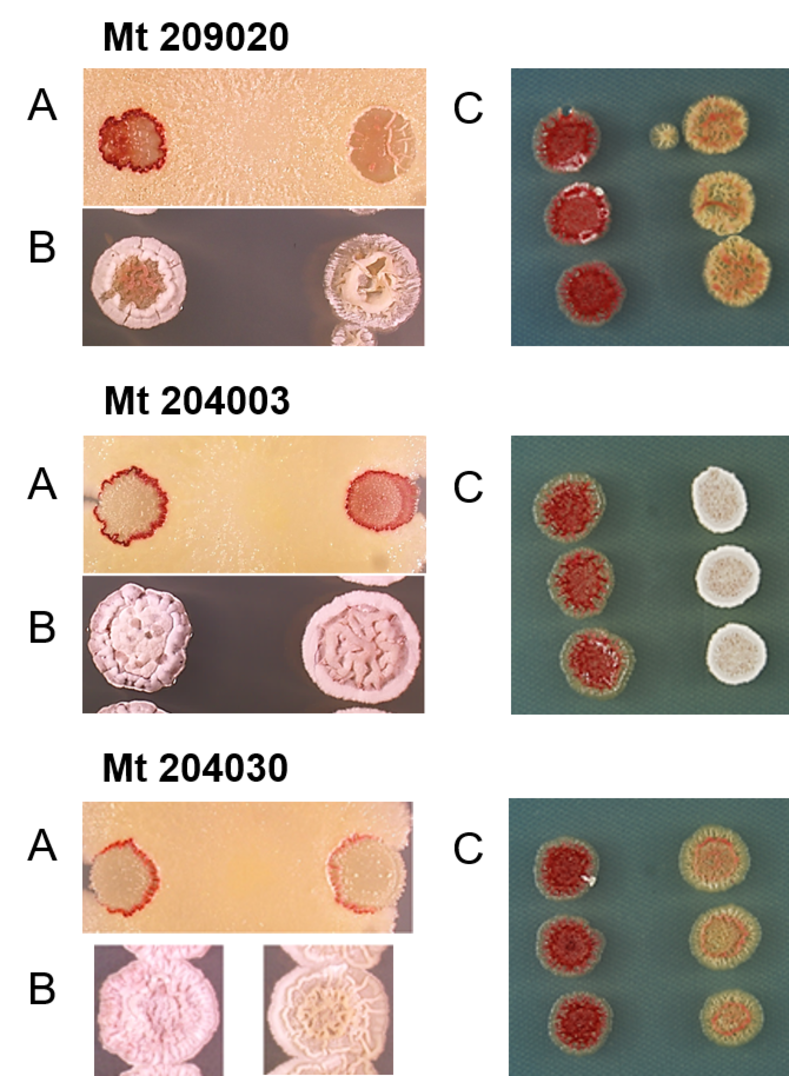

Supplement: S4 Fig — A: dual culture of strain JCM4020 wild type (Wt) or respective Mutant (Mt) with Tsukamurella pulmonis (Tp) grown on YGGS medium, day 5th. B: Wt and Mt grown on Bennett’s maltose, day 11th. C: Wt and Mt grown on Bennett’s maltose+1% NaCl, day 6th. (TIF) [file pone.0270379.s004.tif]

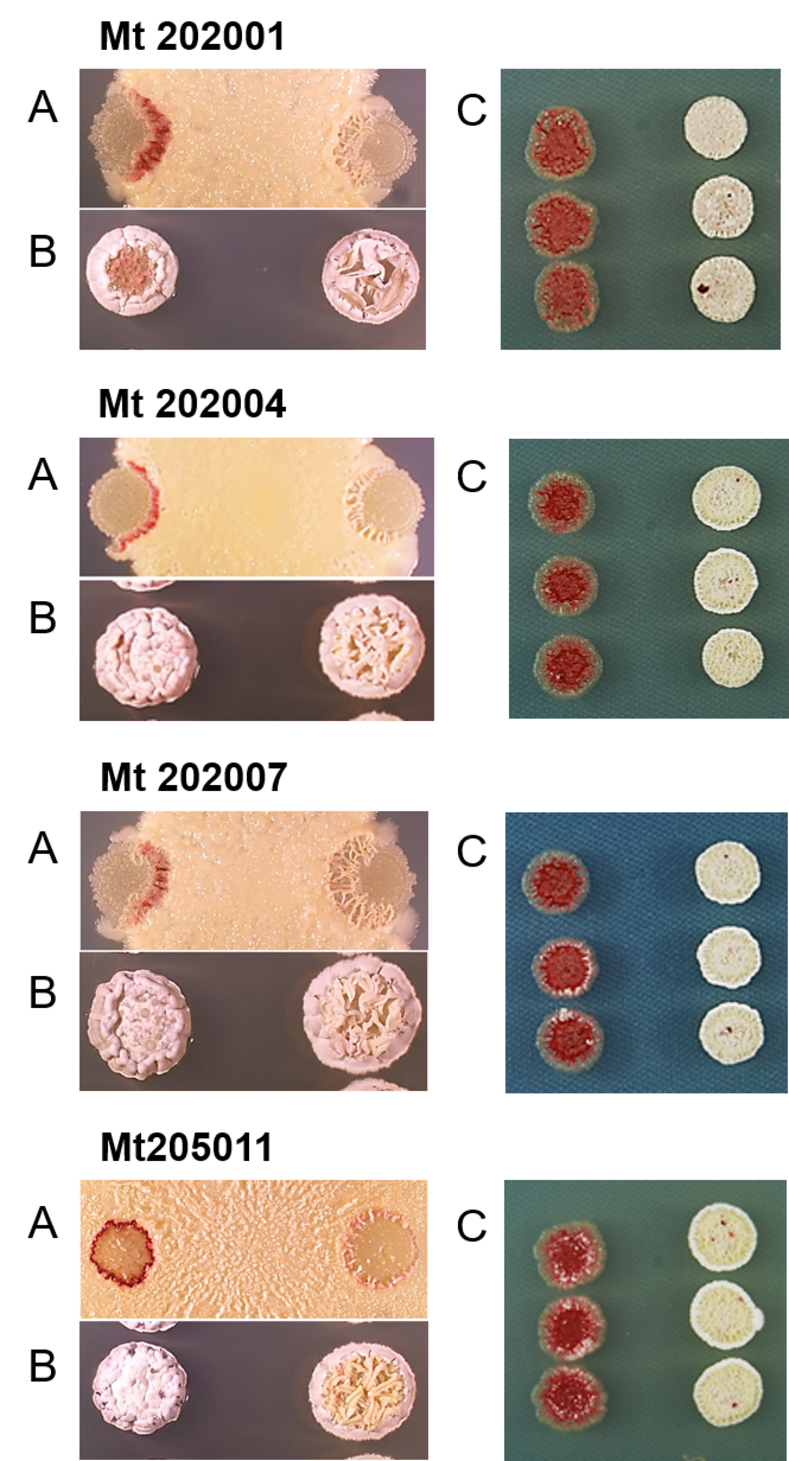

Supplement: S5 Fig — A: dual culture of strain JCM4020 wild type (Wt) or respective Mutant (Mt) with Tsukamurella pulmonis (Tp) grown on YGGS medium, day 5th. B: Wt and Mt grown on Bennett’s maltose, day 11th. C: Wt and Mt grown on Bennett’s maltose+1% NaCl, day 6th. (TIF) [file pone.0270379.s005.tif]

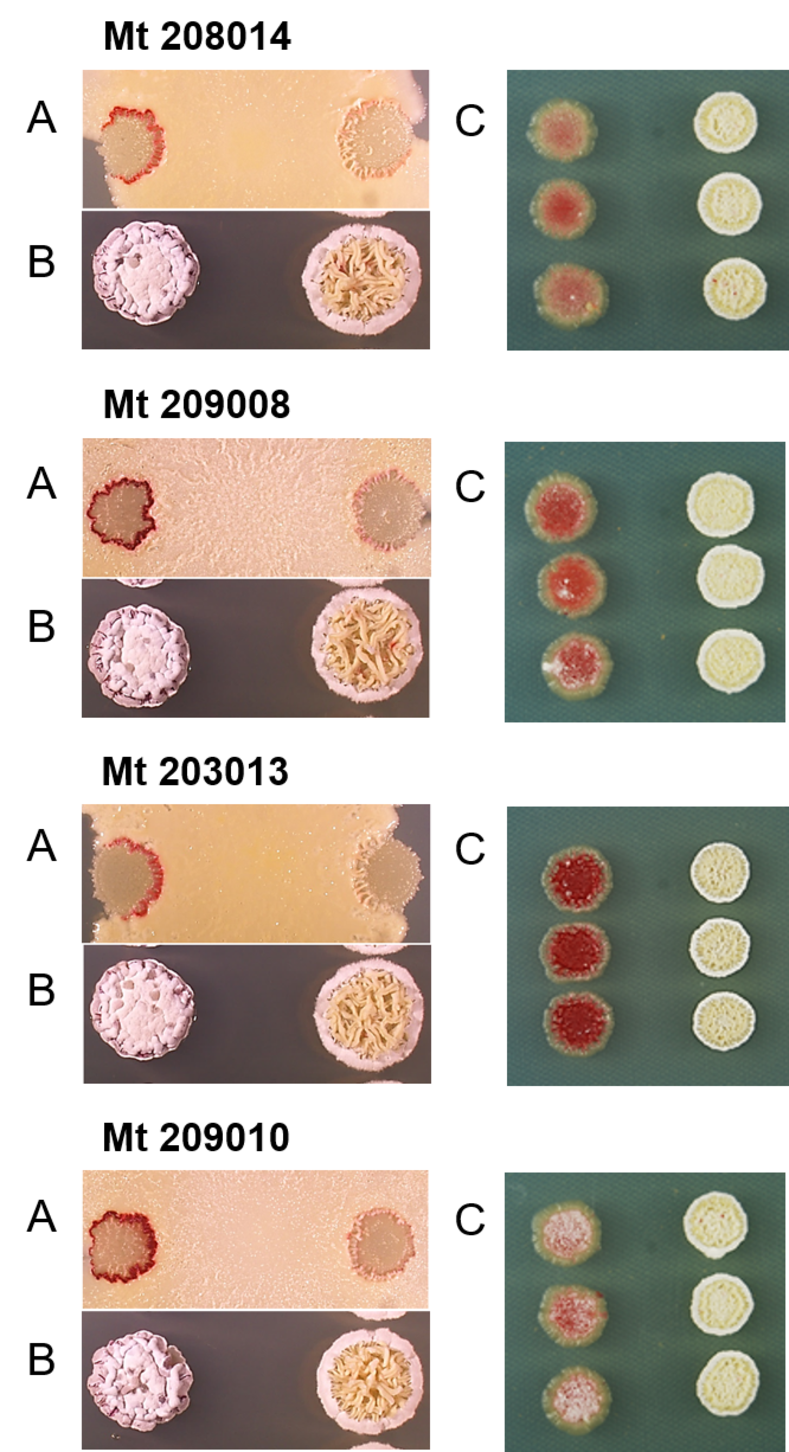

Supplement: S6 Fig — A: dual culture of strain JCM4020 wild type (Wt) or respective Mutant (Mt) with Tsukamurella pulmonis (Tp) grown on YGGS medium, day 5th. B: Wt and Mt grown on Bennett’s maltose, day 11th. C: Wt and Mt grown on Bennett’s maltose+1% NaCl, day 6th. (TIF) [file pone.0270379.s006.tif]

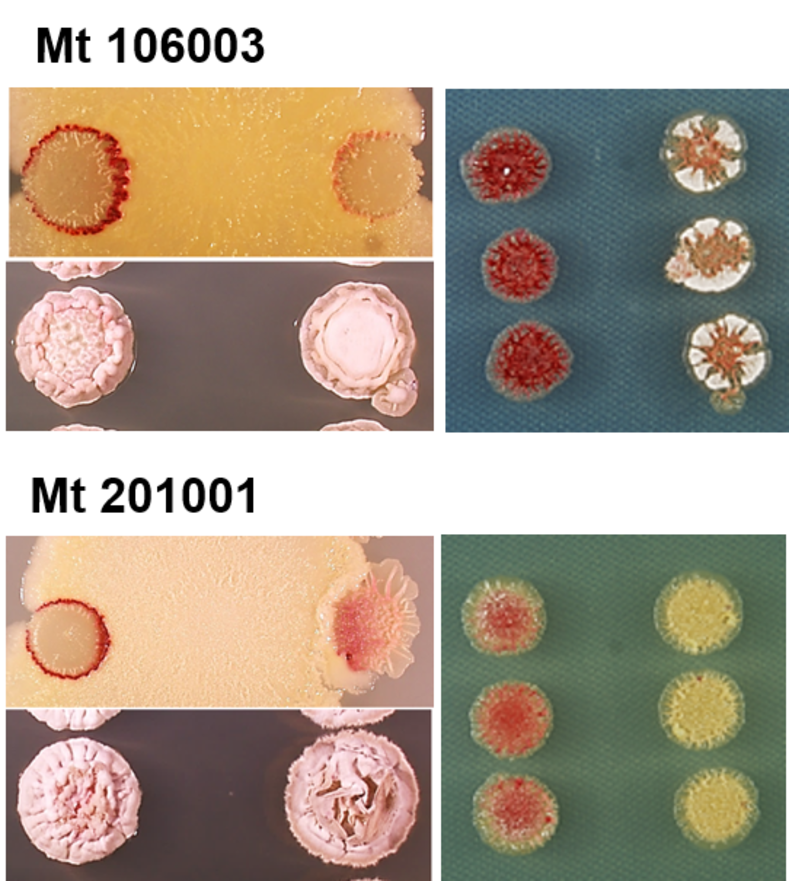

Supplement: S7 Fig — A: dual culture of strain JCM4020 wild type (Wt) or respective Mutant (Mt) with Tsukamurella pulmonis (Tp) grown on YGGS medium, day 5th. B: Wt and Mt grown on Bennett’s maltose, day 11th. C: Wt and Mt grown on Bennett’s maltose+1% NaCl, day 6th. (TIF) [file pone.0270379.s007.tif]

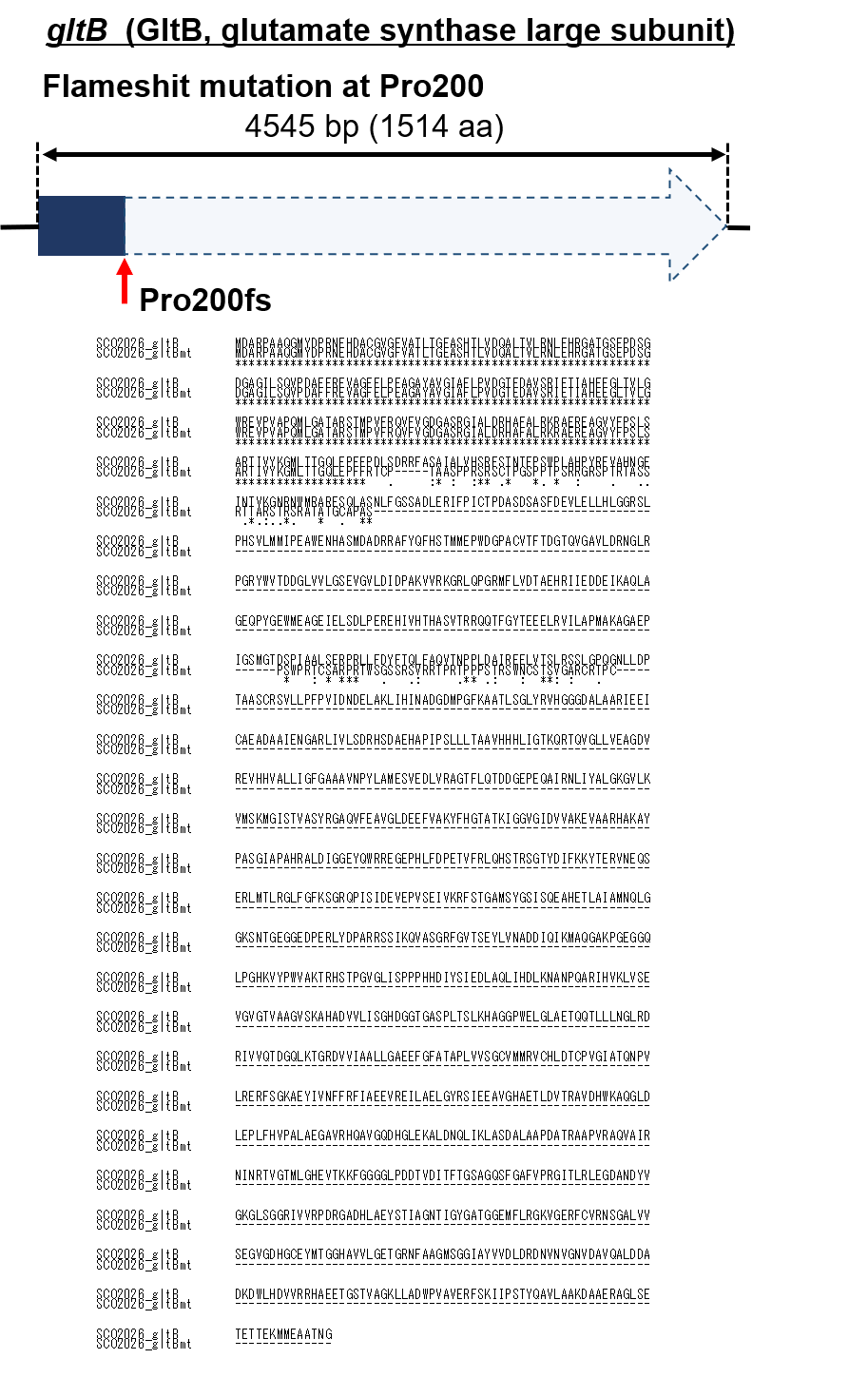

Supplement: S8 Fig — (TIF) [file pone.0270379.s008.tif]

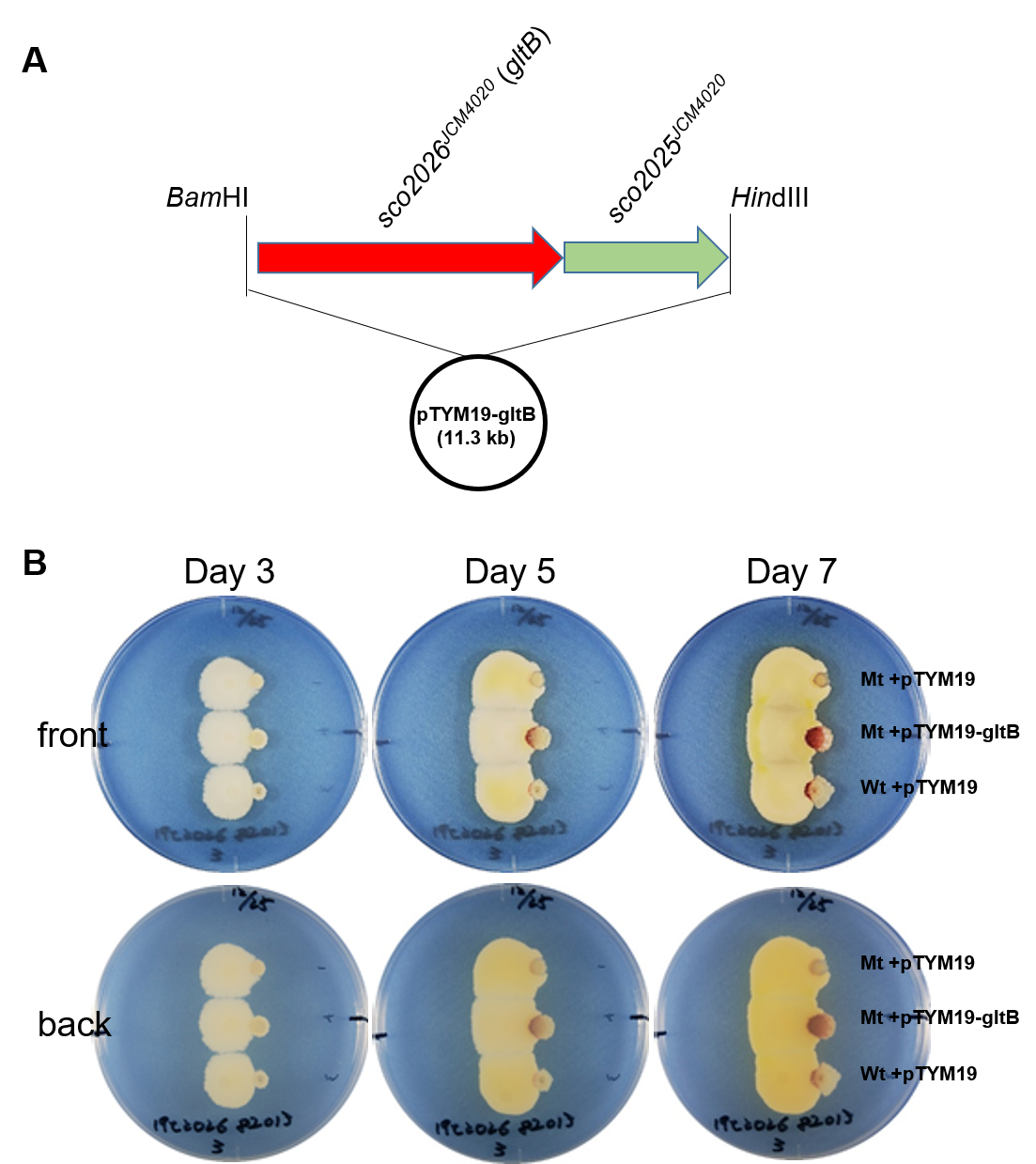

Supplement: S9 Fig — (TIF) [file pone.0270379.s009.tif]

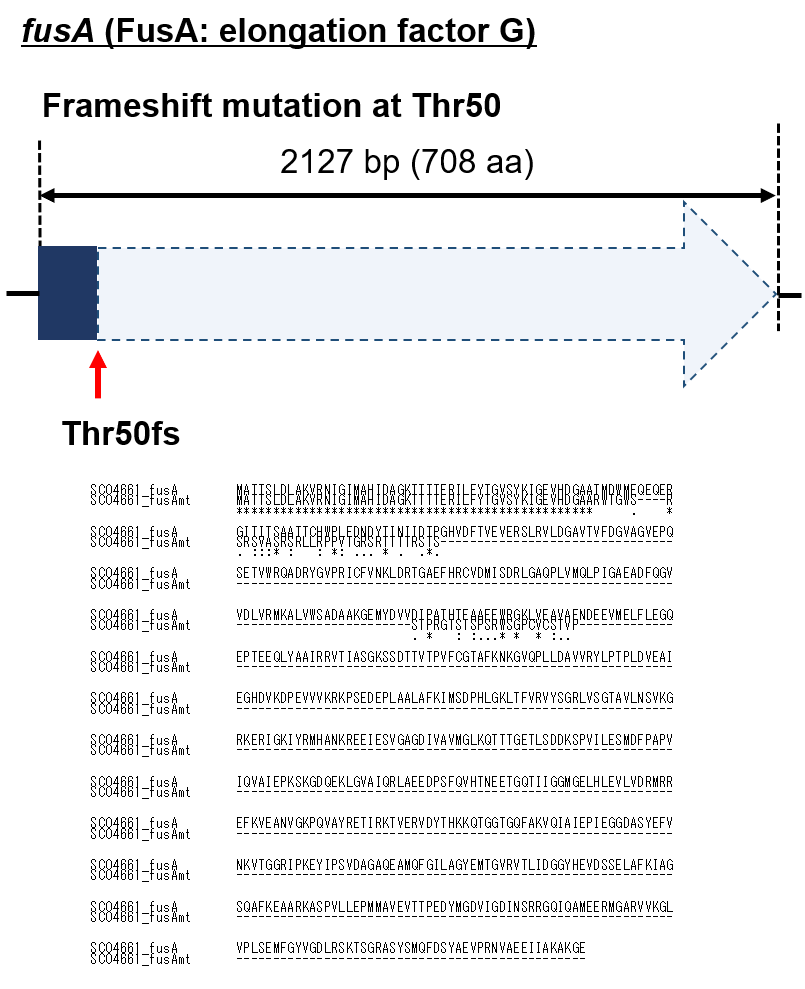

Supplement: S10 Fig — (TIF) [file pone.0270379.s010.tif]

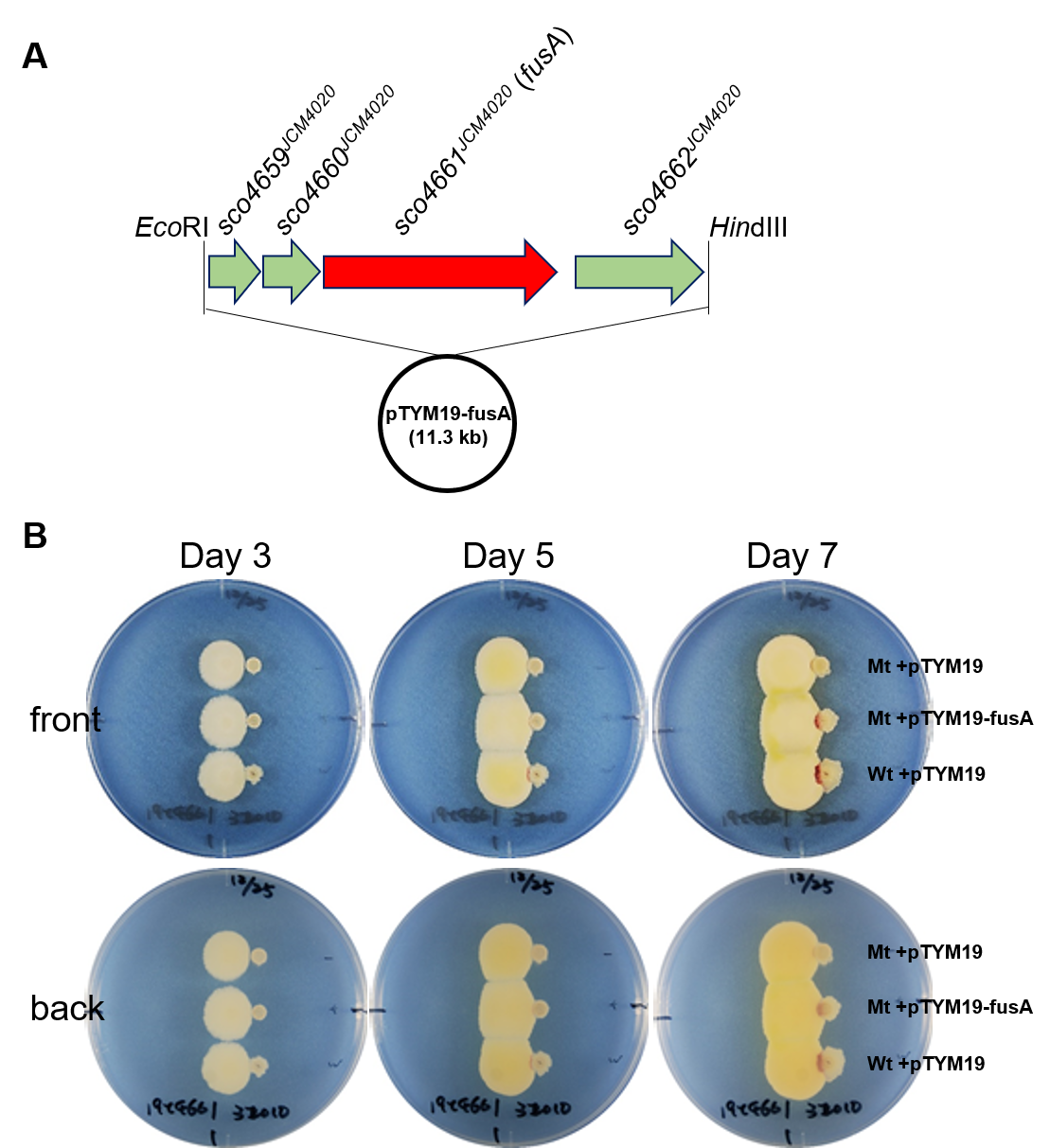

Supplement: S11 Fig — (TIF) [file pone.0270379.s011.tif]

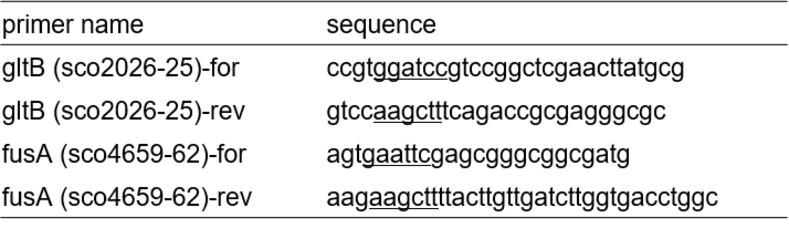

Supplement: S1 Table — (TIF) [file pone.0270379.s012.tif]

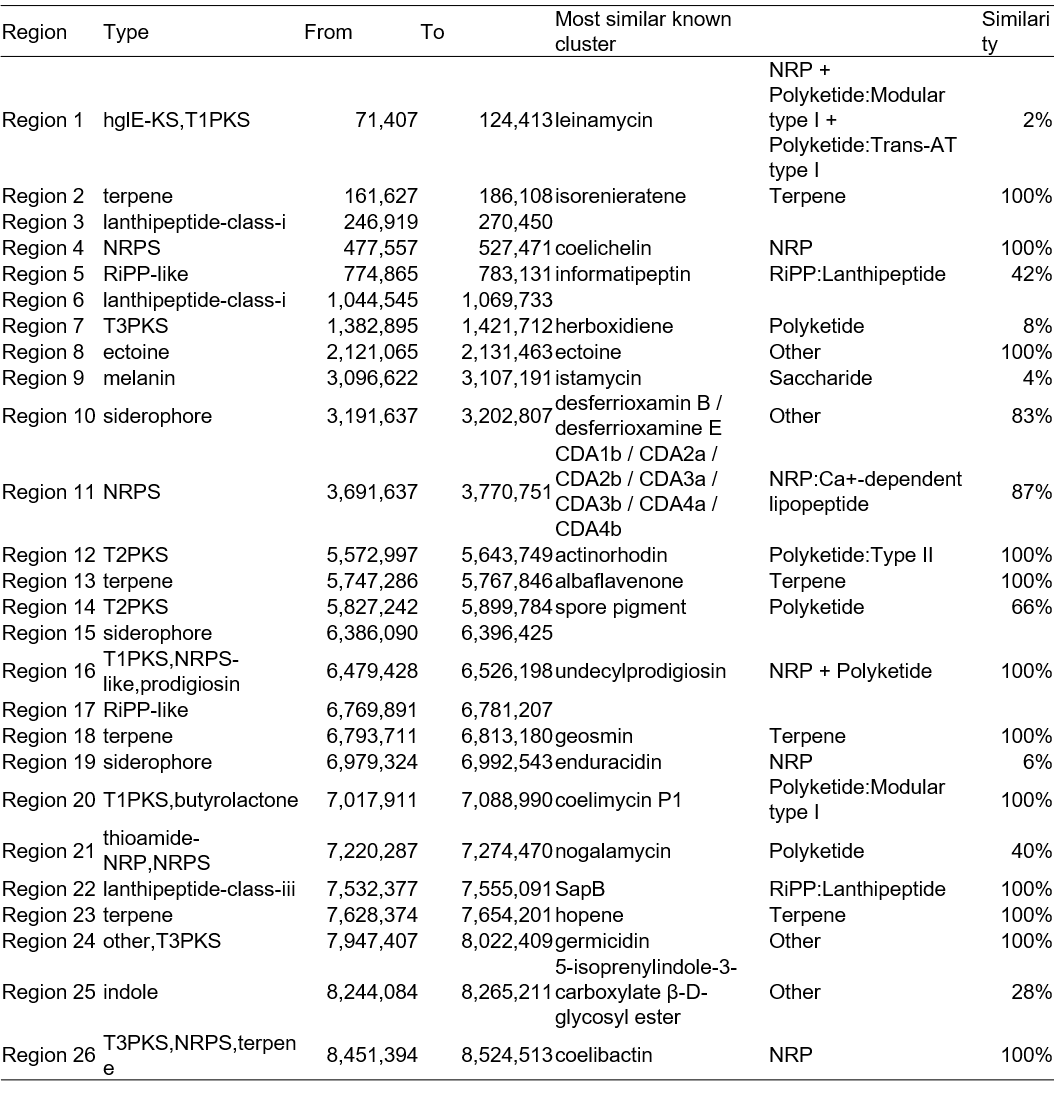

Supplement: S2 Table — (TIF) [file pone.0270379.s013.tif]
